# Understanding the Dermoscopic Patterns of Basal Cell Carcinoma Using Line-Field Confocal Tomography

**DOI:** 10.3390/tomography10060063

**Published:** 2024-05-22

**Authors:** Lorenzo Barbarossa, Martina D’Onghia, Alessandra Cartocci, Mariano Suppa, Linda Tognetti, Simone Cappilli, Ketty Peris, Javiera Perez-Anker, Josep Malvehy, Gennaro Baldino, Caterina Militello, Jean Luc Perrot, Pietro Rubegni, Elisa Cinotti

**Affiliations:** 1 Dermatology Unit, Department of Medical, Surgical and Neurological Sciences, University of Siena, 53100 Siena, Italylinda.tognetti@dbm.unisi.it (L.T.); pietro.rubegni@gmail.com (P.R.); elisa.cinotti@unisi.it (E.C.); 2 Department of Dermatology, Hôpital Erasme, HUB, Université Libre de Bruxelles, 1070 Brussels, Belgium; 3 Department of Dermatology, Sacred Heart Catholic University, 00168 Rome, Italy; simo.cappilli@gmail.com (S.C.);; 4 Melanoma Unit, Hospital Clinic Barcelona, University of Barcelona, 08036 Barcelona, Spain; 5 CIBER de Enfermedades Raras, Instituto de Salud Carlos III, 28029 Barcelona, Spain; 6 Department of Biomedical and Dental Sciences and Morphofunctional Imaging, University of Messina, 98122 Messina, Italy; 7 Department of Pathological Anatomy, University of Siena, 53100 Siena, Italy; 8 Department of Dermatology, University Hospital of Saint-Etienne, 42055 Saint-Etienne, France

**Keywords:** LC-OCT, optical coherence tomography, basal cell carcinoma, imaging, dermoscopy

## Abstract

Basal cell carcinoma (BCC) is the most frequent malignancy in the general population. To date, dermoscopy is considered a key tool for the diagnosis of BCC; nevertheless, line-field confocal optical coherence tomography (LC-OCT), a new non-invasive optical technique, has become increasingly important in clinical practice, allowing for in vivo imaging at cellular resolution. The present study aimed to investigate the possible correlation between the dermoscopic features of BCC and their LC-OCT counterparts. In total, 100 histopathologically confirmed BCC cases were collected at the Dermatologic Clinic of the University of Siena, Italy. Predefined dermoscopic and LC-OCT criteria were retrospectively evaluated, and their frequencies were calculated. The mean (SD) age of our cohort was 65.46 (13.36) years. Overall, BCC lesions were mainly located on the head (49%), and they were predominantly dermoscopically pigmented (59%). Interestingly, all dermoscopic features considered had a statistically significant agreement with the LC-OCT criteria (all *p* < 0.05). In conclusion, our results showed that dermoscopic patterns may be associated with LC-OCT findings, potentially increasing accuracy in BCC diagnosis. However, further studies are needed in this field.

## 1. Introduction

Basal cell carcinoma (BCC) is the most common malignant cancer in humans, with an increasing incidence worldwide [1,2]. It is primarily associated with chronic sun exposure, mostly arising on sun-damaged skin [3]. Generally, BCC is a slowly growing tumor that rarely metastasizes. Nevertheless, it can be locally invasive and destructive, especially when its diagnosis is delayed [4].

Dermoscopy has become an indispensable tool for the identification of BCC in real-life practice, enabling early detection and its discrimination from other skin lesions [5]. To date, dermoscopic patterns of BCC have been widely described in the literature [6]. They include the absence of a pigmented network combined with other criteria, such as ulceration, blue–gray ovoid nests, globules, and maple-leaf-like and spoke-wheel areas [6,7]. However, BCC has a wide variety of manifestations, and proper diagnosis may be challenging, especially when it is based on clinical and dermoscopic information alone [8].

Currently, histology is well accepted as the gold-standard assessment tool for BCC subtyping [9]. Nevertheless, new non-invasive optical techniques have proven to play an increasingly central role in routine skin cancer diagnosis, sparing patients from undergoing useless biopsies [10,11]. Among these technologies, line-field confocal optical coherence tomography (LC-OCT) has demonstrated excellent performance in real-life practice not only in detecting BCC but also in monitoring the disease course after medical treatments when surgery is not required [12].

In particular, LC-OCT has gained increased popularity since it offers a real-time 3D visualization of skin images in a vertical and horizontal view, combining the advantages of optical coherence tomography (OCT) and reflectance confocal microscopy (RCM), thus overcoming their limitations regarding spatial resolution, penetration, and image orientation [13]. Interestingly, LC-OCT provides in vivo images of the entire skin lesions at a cellular resolution close to histological examination [14]. Also, it allows for a real-time dermoscopic/LC-OCT correlation due to the integration of a dermoscope into the LC-OCT device. Given this background, the purpose of our paper was to investigate the possible correspondence between BCC dermoscopic features and their LC-OCT counterparts.

## 2. Materials and Methods

A retrospective observational monocentric study was performed. The database of the LC-OCT device (DAMAE Medical, Paris, France) of the Dermatology Department of the University Hospital of Siena was examined. In total, 100 BCC lesions with LC-OCT images and histological examination (either of a complete excision or skin biopsy) were consecutively selected between September 2021 and October 2023. LC-OCT images were acquired by an expert in skin imaging with more than 10 years of experience in dermoscopy and OCT (E.C.).

The LC-OCT system consists of a portable probe connected to a central unit and a display, which offers a 10 frames/second acquisition rate, an axial resolution of 1.2 μm, a lateral resolution of 1.3 μm, a scanning depth of 500 μm, and, finally, a lateral field of view of 1.2 mm. To guarantee that the refractive indices are matched, a drop of paraffin oil is applied between the lesion and the LC-OCT camera lens. Vertical sections and/or 3D images can be acquired.

Dermoscopic images are obtained through an integrated camera inside the probe. It is important to highlight that the videodermoscope integrated with LC-OCT has a 2.5 × 2.5 mm field of view and 5-micron resolution, allowing for dermoscopic images with higher magnification compared to traditional dermoscopy, which should not be misinterpreted as low image quality. Generally, it serves not diagnostic purposes but rather correlates lesion dermoscopy with LC-OCT images in real time, aiding clinicians during the examination. Finally, a marker inside the dermoscopic image constantly highlights the corresponding LC-OCT field of view during the examination.

In our study, images with a confirmed histological diagnosis were re-evaluated together by two dermatologists (L.B. and M.D.) with intermediate LC-OCT experience (about 12 months) and one expert dermatologist (E.C., >10 years of LC-OCT experience). All videos and vertical and/or 3D images available for each lesion were considered.

According to the current literature, the following dermoscopic patterns were assessed: ulceration, erosion, maple-leaf figures, blue–gray globules, large blue–gray ovoid nest, telangiectasia and arborizing vessels, multiple in-focus blue–gray dots (peppering, <0.1 mm), pink–white areas, blue-whitish veils, and milia-like cysts. Based on our experience, for each dermoscopic criterion, we assumed the presence of possible LC-OCT features. The LC-OCT images were described using previously proposed terms as follows: ulceration and erosion, tumor lobules with maple-leaf shapes, roundish medium-reflective tumor lobules, telangiectasia or arborizing vessels, hyper-reflective roundish small areas inside tumor lobules, non-pigmented/medium-reflective lobules, hyper-reflective lobules in the entire dermis, and milia-like cysts.

Descriptive statistics included the mean and standard deviation (SD) for quantitative variables, whereas frequency and percentage were reported for categorical variables. To compare dermoscopic features with those of LC-OCT, the Gwet AC1 concordance index and the correct classification rate (CCR) were estimated [15,16]. *p* < 0.05 was considered statistically significant. All analyses were performed using R software version 4.1.0 (R Foundation for Statistical Computing, Vienna, Austria).

## 3. Results

The demographic and clinical characteristics of all the lesions evaluated are summarized in Table 1. In total, 53 (53%) subjects were male. The mean (±SD) age at diagnosis was 65.46 (13.36) years old. Lesions were mainly located on the head (49%), followed by the legs (12%), back (11%), thorax (10%), arm (6%), ears (6%), abdomen (4%), and neck (2%). BCCs histologically confirmed as superficial represented 54% of the sample, followed by nodular (n = 20), infiltrating (n = 16), micronodular (n = 5), and basosquamous (n = 5) subtypes. Additionally, dermoscopic pigmented BCCs accounted for 59% of the entire cohort.

Telangiectasia or arborizing vessels (51%), blue–gray globules (40%), and multiple blue–gray dots (12%) were the most common patterns detected in the dermoscopic images, followed by maple-leaf figures (12%), large blue–gray ovoid nests (10%), and pink–white areas (10%). Considering LC-OCT evaluations, the most commonly observed aspects were telangiectasia or arborizing vessels (39%), followed by medium-reflective tumor lobules detached from the epidermis with variable hyper-reflective spots (36%) and hyper-reflective roundish small areas inside tumor lobules (13%), which corresponded to multiple blue–gray globules and dots detected when using dermoscopy, respectively.

Overall, the prevalences of the dermoscopic and LC-OCT criteria assumed were similar with both methods (all *p* > 0.05), and the concordance between dermoscopic and LC-OCT patterns was remarkably high for all aspects evaluated (all *p* < 0.001) (Table 2).

Erosion and ulceration, maple-leaf figures identified under LC-OCT as medium-reflective lobules with maple-leaf shapes attached to the epidermis, and the blue-whitish veil which corresponded to hyper-reflective lobules in the dermis, were present in the same percentage of cases with both techniques (7%, 12% and 9%, respectively), showing a high level of concordance (all *p* < 0.001).

Blue–gray globules, blue–gray ovoid nests, multiple blue–gray dots, pink–white areas, and milia-like cysts were more visible under dermoscopy than LC-OCT (Table 2), with a good grade of concordance (0.96, 0.99, 0.95, 0.98, and 0.90, respectively). Finally, the identification of telangiectasia and arborizing vessels was easier with dermoscopy (51%) compared to LC-OCT (39%), with a slightly lower concordance compared to the other criteria (0.76).

## 4. Discussion

Dermoscopy is currently considered a key tool and a routine method for BCC detection. It provides a macroscopic view of cutaneous lesions, allowing for the discrimination of BCC from other skin tumors [17]. Currently, dermoscopic BCC criteria have been updated and enriched several times [18]. Interestingly, their association with histopathologic counterparts has been extensively described in the literature, providing an ideal link to enhance clinical–pathological correlations and improve BCC diagnosis [19,20]. However, histopathology generally explores just a few slices of skin lesions; thus, a possible correlation with dermoscopy could be lost during the processing phase. Moreover, defining a direct and accurate dermoscopic–histopathologic relationship could be complicated by the fact that dermoscopic horizontal images need to be compared with the traditional histopathological vertical sections. Interestingly, several studies have explored the association between non-invasive RCM and histopathology, facing similar difficulties. Indeed, RCM allows for the examination of cytological and architectural elements in horizontal images, while histopathological ones are vertical. Nevertheless, Perino et al. [21] recently described the concordance between those methods in BCC diagnosis, finding a good correlation between them.

LC-OCT is an innovative non-invasive optical imaging technique that allows for the identification of BCC in vertical, horizontal, and three dimensions, offering a real-time correlation with dermoscopic images [13]. Indeed, LC-OCT is integrated with a dermoscope with the concurrent colocalization of dermoscopic and LC-OCT images. Therefore, while vertical scans can be directly comparable to histological images, the horizontal ones can be easily related to dermoscopy.

Currently, LC-OCT has been gaining growing attention because it provides detailed microscopic insight into skin structures at a cellular level, thus playing a central role in the identification of BCC [22]. Recently, Gust et al. [23] demonstrated that the accuracy of LC-OCT, especially in equivocal BCC, was higher compared to dermoscopy and that, surprisingly, the association between those techniques reached an accuracy similar to the gold-standard histology [23]. Finally, our group confirmed these results via two real-life studies conducted over three years, showing that LC-OCT had a higher specificity, sensitivity, and accuracy than dermoscopy alone [24,25].

Given this background, the goal of the present study was to explore the correlation between dermoscopic structures and LC-OCT findings in BCC.

Overall, 100 BCC lesions were included in this study. The mean age at diagnosis was 65.46 years, and 53% of the entire cohort were males. BCCs were mainly located on the head and neck (57%), followed by the trunk (25%) and the inferior extremities (12%), thus confirming that LC-OCT is a particularly useful tool for BCCs located on the face, helping us to reduce the number of skin biopsies performed in this critical area. We found that 59% of BCCs were clinically pigmented, while 54% of the entire sample was classified as superficial via histopathology, followed by nodular (n = 20), infiltrating (n = 16), micronodular (n = 5), and basosquamous (n = 5) subtypes.

Generally, the most distinct feature of BCC detected via LC-OCT is the presence of tumor lobules, which are aggregates of basaloid cells growing into the dermis [26]. They are typically characterized by the presence of a gray core with a laminated structure parallel to the epidermis corresponding to dense cellularity (“millefeuille pattern”) [26]. Tumor lobules are often surrounded by a middle dark rim (clefting), corresponding to peritumoral mucin deposition, and an outer bright rim, characterized by a higher reflectivity, probably due to tumor island compression on the collagen fibres [26].

Based on previous works [27] and our experience, we attempted to assess the association between dermoscopy and LC-OCT using ten-point criteria. Overall, we found that most dermoscopic aspects evaluated were also appreciable under LC-OCT, and no statistically significant differences were found between them (*p* > 0.005). Moreover, an excellent correlation was found among all postulated criteria (*p* < 0.0001).

We observed that erosion and ulceration, resulting from the complete or partial absence of the epidermis (erosion) and dermis (ulceration) [28], appeared, via LC-OCT, as dark areas with sharp borders and irregular contours filled with cellular debris. These findings were either limited to the superficial skin or involved the entire epidermis and dermis layer. In our experience, ulceration and erosion were equally appreciable under dermoscopy and LC-OCT (7%), with a total dermoscopic/LC-OCT correlation (*p* < 0.001). Notably, erosion and ulceration were evaluated together because differentiating the level of skin loss could be challenging with both imaging techniques.

**Figure 1:**
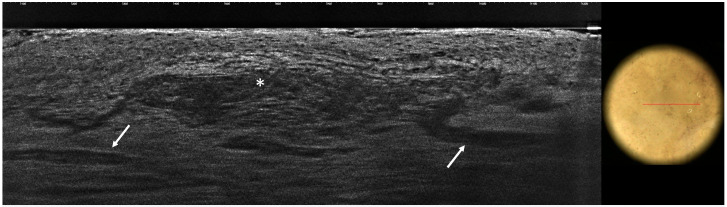
Superficial basal cell carcinoma: LC-OCT image and dermoscopic correlation. LC-OCT examination reveals the presence of lobules with an inner gray core featuring the peculiar *millefeuille* pattern (white asterisks). Blood vessels are visualized as well-defined, hyporeflective elongated structures of various sizes localized within the dermis and next to the lobules (white arrows).

Considering vascular patterns, they were visible via dermoscopy as linear telangiectasia or arborising vessels, compared, via LC-OCT, to roundish or elongated dark hyporeflective structures within the dermis, depending on the orientation of their sections [29] (Figure 1). Since the telangiectasia or arborising vessels observed in the dermoscopic images (51%) were not always detected under LC-OCT (39%), we hypothesized that this was probably due to the lack of BCC 3D images, which may be particularly useful in the detection of vascular structures. In our work, we were unable to distinguish fine linear telangiectasia from arborizing vessels using LC-OCT. However, given their different morphologies when using dermoscopy, we can speculate that in-focus arborizing vessels could correspond to enlarged vessels pushed toward the surface by the underlying BCC tumor lobules via LC-OCT, whereas short, fine, linear vessels could correspond to vessels located under the superficial lobules appended to the epidermis.

**Figure 2:**
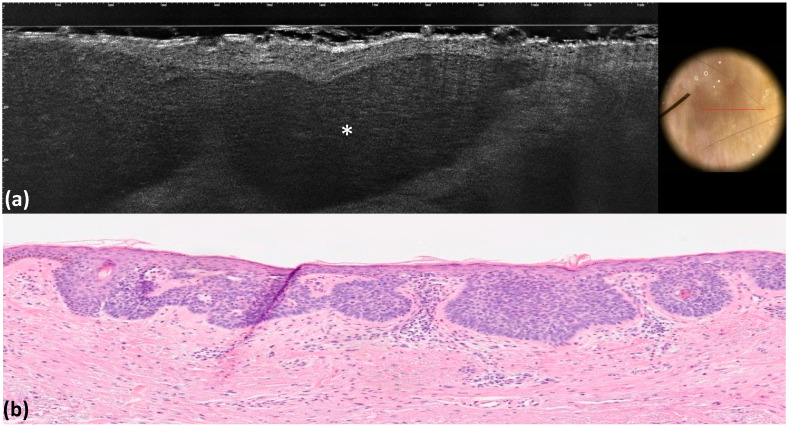
Superficial basal cell carcinoma: LC-OCT image and dermoscopic correlation (**a**). Dermoscopy shows pink–white areas, whereas LC-OCT examination reveals the presence of medium-reflective superficial lobules attached to the epidermis with a *millefeuille* pattern (white asterisks) (**a**). Histological image, hematoxylin–eosin (x4) (**b**).

Pink–white areas represent a common dermoscopic feature of non-pigmented BCC, which dermoscopically appears with pink and milky white areas [7] (Figure 2). Curiously, those structures were not always appreciable in our dermoscopic evaluations. This was probably because the dermoscope coupled with the LC-OCT device provides images with a slightly altered color compared to the normal skin, thus affecting the evaluation of this criteria in most of our non-pigmented BCCs. Overall, we observed that pink–white areas were visible under dermoscopy in 10% of cases, while their corresponding LC-OCT counterparts (medium-reflective tumor lobules) were detected in 8% of cases.

In nine and five cases, respectively, we observed milia-like-cysts when performing dermoscopy and LC-OCT. Generally, milia-like-cysts appear as yellowish, roundish structures when using dermoscopy, and even though they are more frequently observed in benign epidermal lesions, their presence should not rule out BCC diagnosis [30].

**Figure 3:**
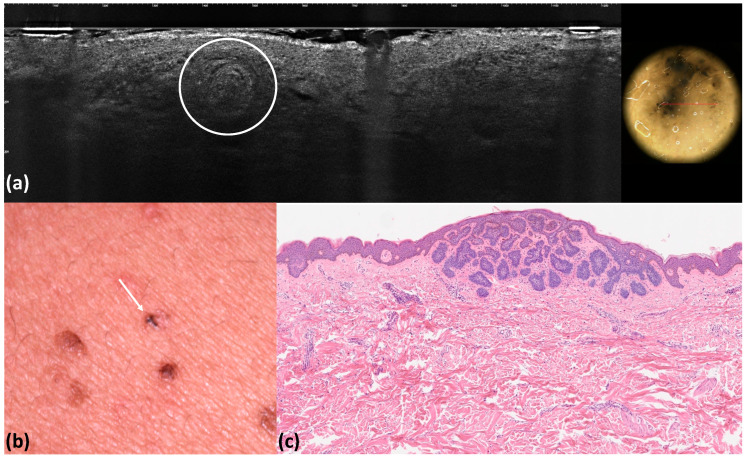
Micronodular basal cell carcinoma: LC-OCT image and its dermoscopic correlation. LC-OCT shows a milia-like cyst characterized by hyper-reflective roundish areas with an onion-like shape (white circle) corresponding to keratin. A white roundish area is visible in the corresponding dermoscopic section (**a**). Clinical image (**b**). Histological image. (**c**) Hematoxylin–eosin (x4).

During LC-OCT, milia-like cysts were visible as small hyper-reflective roundish areas filled with amorphous material with a concentric organization, corresponding to keratin with an onion-like shape (Figure 3). To conclude, considering both pink–white areas and milia-like cysts, we found a statistically significant correlation index between dermoscopy and LC-OCT (all *p* < 0.001).

**Figure 4:**
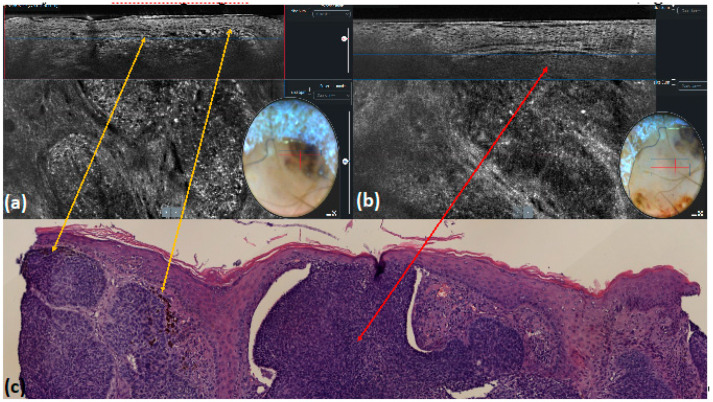
Nodular basal cell carcinoma: LC-OCT images and their dermoscopic correlation with pigmented and non-pigmented areas of the lesion (**a**,**b**). Hyper-reflective roundish small areas corresponding to melanophages are visible under LC-OCT inside the lobules in the part of the tumor that is pigmented when using dermoscopy (**a**). Histological correlation shows numerous melanophages (yellow arrows) (**c**). Medium-reflective roundish lobules, clefting, and hyper-reflective stroma are visible under LC-OCT in the adjacent area without pigment when using dermoscopy (**b**). Histological correlation of the non-pigmented lobule (red arrow) (**c**).

Similar to non-pigmented, pigmented BCCs are characterized via LC-OCT by tumor lobules, constituted by medium-reflective laminated roundish structures. In addition, bright roundish cells, probably corresponding to activate melanocytes or melanophages, can be observed within the lobules [26] (Figure 4).

Interestingly, the dermoscopic diagnosis of pigmented BCC seems to have higher accuracy compared to non-pigmented subtypes due to the presence of characteristic dermoscopic criteria [31]. In our cohort, pigmented BCC accounted for 59%. In particular, the following dermoscopic patterns were considered: maple-leaf-like areas, blue–gray ovoid nest, globules, dots, and blue-whitish veils.

**Figure 5:**
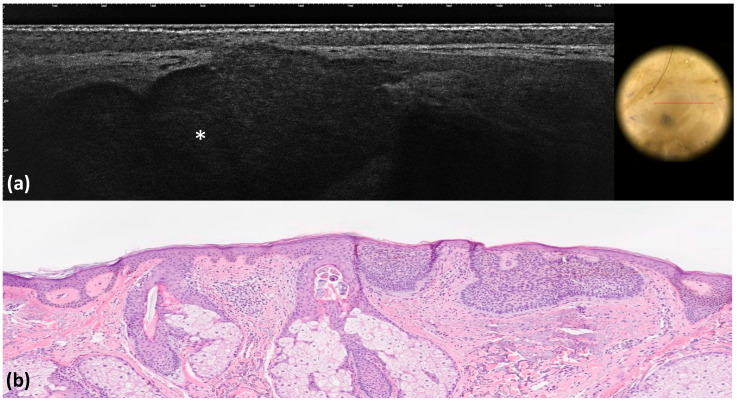
Superficial basal cell carcinoma: LC-OCT image and dermoscopic correlation. Dermoscopy shows maple-leaf-like areas, which, via LC-OCT, appear as medium-reflective lobules with a maple-leaf shape attached to the epidermis (white asterisk) (**a**). Histological image, hematoxylin–eosin (x4) (**b**).

Generally, maple-leaf-like areas are described as bulbous extensions connected to a common base, with a leaf-like pattern at the periphery of the tumor that never arises from a pigmented network or adjacent confluent pigmented areas [6]. During LC-OCT evaluation, these structures were observed as medium-reflective lobules with maple-leaf shapes attached to the epidermis (Figure 5). From our results, maple-leaf-like areas corresponding to medium-reflective lobules with maple-leaf shapes were appreciable in 12% of cases considering both dermoscopy and LC-OCT, thus showing a high level of concordance (*p* < 0.001). Although they were not evaluated in our study, Palmisano et al. [27] revealed a correlation between negative maple-leaf shape areas and deep hypopigmented tumor lobules.

**Figure 6:**
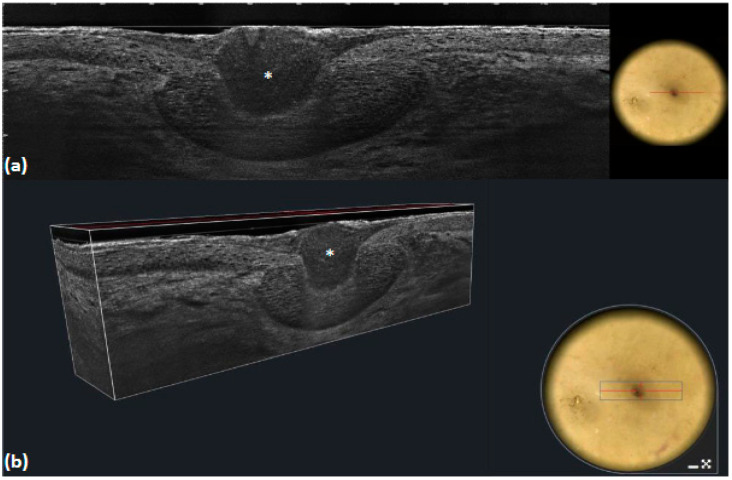
Superficial basal cell carcinoma: LC-OCT image and its dermoscopic correlation (**a**,**b**). Dermoscopy shows a concentric structure, which on vertical (**a**) and 3D (**b**) LC-OCT images appears as a medium-reflective lobule connected to the epidermis around a central dilated hair follicle (white asterisk).

Interestingly, we observed two concentric structures when using dermoscopy, which corresponded to the hyper-reflective superficial island of BCC connected to the epidermis around the central dilated hair follicles detected via LC-OCT (Figure 6).

Blue–gray ovoid nests are typically described as well-circumscribed, confluent, or near-confluent pigmented ovoid or elongated areas, larger than globules, and not intimately connected to pigmented tumor body [8]. Via LC-OCT, we observed that they mainly consisted of medium-reflective tumor lobules detached from the epidermis, with variable hyper-reflective spots.

In addition, we noted that, when using LC-OCT, globules were not only associated with deep medium-reflective tumor lobules and variable hyper-reflective spots but also with hyporeflective roundish areas, suggestive of necrotic areas inside the tumors. In total, we found 10% of blue–gray ovoid nests and 40% of blue–gray globules compared to 9% and 36% of their LC-OCT counterparts, respectively. Overall, the grade of concordance was high (0.99 and 0.96, respectively).

**Figure 7:**
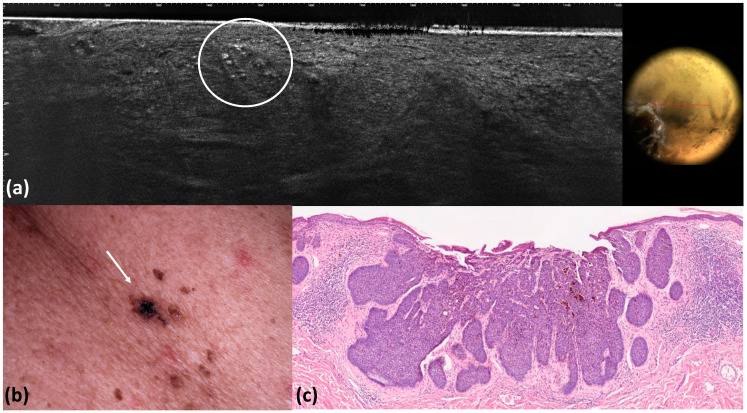
Nodular basal cell carcinoma: LC-OCT image and its dermoscopic correlation (**a**). Dermoscopy shows multiple blue–gray dots referring to roundish hyper-reflective areas within the lobule, which, when using LC-OCT, correspond to melanophages (white circle) (**a**). Clinical image (**b**). Histological image. (**c**) Hematoxylin–eosin (x4).

**Figure 8:**
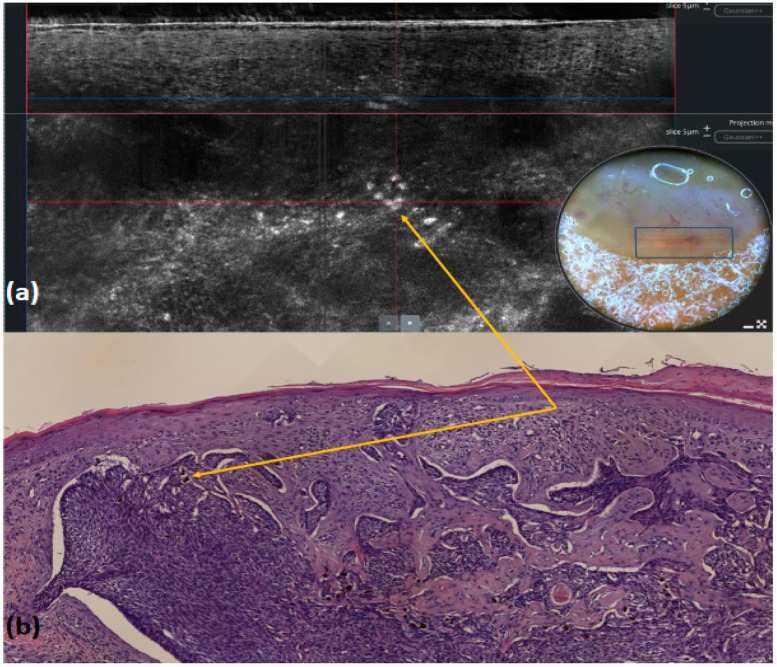
Nodular basal cell carcinoma: LC-OCT image and its dermoscopic (**a**) and histologic (**b**) correlation (yellow arrows). Dermoscopy shows multiple blue–gray dots that appear as roundish hyper-reflective areas within the lobule, which, (**a**) when using LC-OCT, correspond to melanophages upon histological examination ((**b**), hematoxylin and eosin staining).

In accordance with current dermoscopic criteria, blue–gray dots observed as well-defined small gray dots [32] were detected in 18% of cases. In 13% of the lesions, they correlated with hyper-reflective roundish small areas inside the tumor lobules when using LC-OCT, probably corresponding to melanocytes, melanophages, or pigmented keratinocytes within the BCC lobules (Figure 7 and Figure 8).

**Figure 9:**
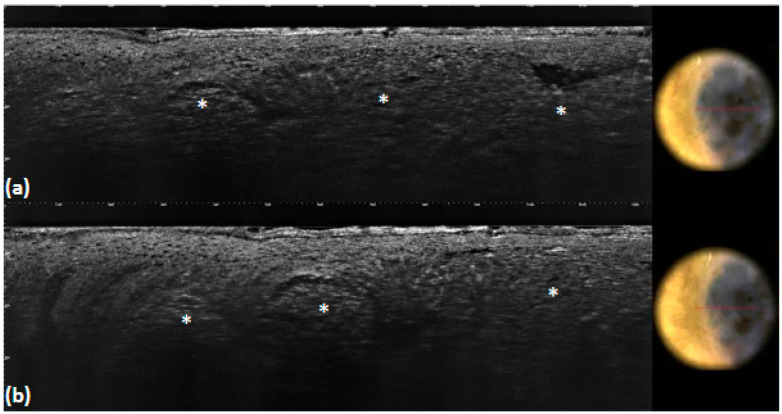
Superficial basal cell carcinoma: LC-OCT images and their dermoscopic correlation (**a**,**b**). Dermoscopy shows a blue-whitish veil, which, when using LC-OCT, corresponds to lobules with hyper-reflective particles inside occupying the entire visible dermis (white asterisks) (**a**,**b**).

Finally, a blue-whitish veil, defined as an irregular structureless area of confluent blue pigmentation with an overlying white “ground-glass” film [20], was represented by hyper-reflective lobules that occupied the entire visible dermis when using LC-OCT (9% via dermoscopy vs. 9% via LC-OCT) (Figure 9).

To the best of our knowledge, this is the first study to assess the possible association between dermoscopic and LC-OCT criteria in BCC. We think that acquiring this knowledge may have the potential to improve clinicians’ diagnostic accuracy, providing further prognostic information that might help them to perform skin biopsies and guide the management of skin cancers.

However, several limitations need to be recognized. Firstly, the retrospective nature of the study is an issue, particularly since both techniques are operator-dependent. Moreover, despite the high number of BCC cases evaluated, only a few visible dermoscopic features were found. In fact, the dermoscopic field that is imaged via LC-OCT is limited (1.2 mm × 0.5 mm in vertical sections and 1.2 mm × 0.5 mm × 0.5 mm in 3D images), and due to the retrospective acquisition of the images, only a few dermoscopic criteria with their corresponding LC-OCT findings were visible per case. Moreover, the quality of the dermoscopic image is lower than that of the conventional dermoscopy, which makes it difficult to identify specific structures, such as pigmented dots and pink–white areas. Lastly, our results may be affected by the small sample size.

## 5. Conclusions

In this study, we investigated the association between the dermoscopic criteria and LC-OCT features of BCC, which may represent a potential guide for physicians to confidently make a very accurate diagnosis. Particularly in the context of clinically equivocal lesions, LC-OCT evaluation could increase the diagnostic performance of distinguishing BCC from other cutaneous lesions compared to dermoscopy only and vice versa. In addition, understanding this relationship might help physicians to work through further steps in the BCC treatment process. However, further prospective studies should be performed to provide further LC-OCT images of the most relevant dermoscopic areas for a large number of cases.

## Figures and Tables

**Table 1:** General demographic, clinical, and histological features of BCC.

	Overall, n (%) (n = 100)
Male, n (%)	53 (53)
Age at diagnosis, mean (SD)	65.46 (13.36)
BCC localization	
Trunk, n (%)	25 (25)
Inferior extremities, n (%)	12 (12)
Superior extremities, n (%)	6 (6)
Head and neck, n (%)	57 (57)
Dermoscopic pigmented BCC, n (%)	59 (59)
BCC histological subtypes, n (%)	
Superficial, n (%)	54 (54)
Nodular, n (%)	20 (20)
Infiltrating, n (%)	16 (16)
Micronodular, n (%)	5 (5)
Basosquamous, n (%)	5 (5)

Legend: BCC, basal cell carcinoma.

**Table 2:** Relative frequencies and agreement between dermoscopy and the LC-OCT criteria of BCC lesions.

		Overall (n = 100)					
Dermoscopy Criteria	LC-OCT Criteria	Dermoscopy	LC-OCT	P	CCR	Concordance	*p* Concordance
Ulceration or erosion	Ulceration or erosion	7 (7.0)	7 (7.0)	1	1	1	<0.001
Maple-leaf figures	Medium-reflective lobules with maple-leaf shapes attached to the epidermis	12 (12.0)	12 (12.0)	1	1	1	<0.001
Blue–gray globules	Roundish medium-reflective tumor lobules detached from the epidermis with variable hyper-reflective spots	40 (40.0)	36 (36.0)	0.662	0.96	0.92	<0.001
Blue–gray ovoid nests	Medium-reflective tumor lobules detached from the epidermis with variable hyper-reflective spots	10 (10.0)	9 (9.0)	1	0.99	0.99	<0.001
Telangiectasia or arborizing vessels	Telangiectasia or arborizing vessels	51 (51.0)	39 (39.0)	0.118	0.88	0.76	<0.001
Multiple blue–gray dots	Hyper-reflective roundish small areas inside tumor lobules	18 (18.0)	13 (13.0)	0.435	0.95	0.93	<0.001
Pink–white areas	Medium-reflective lobules	10 (10.0)	8 (8.0)	0.805	0.98	0.98	<0.001
Blue-whitish veils	Hyper-reflective lobules in the entire dermis	9 (9.0)	9 (9.0)	1	1	1	<0.001
Milia-like cysts	Milia-like cysts	9(9.0)	5 (5.0)	0.406	0.90	0.89	<0.001

Legend: CCR, correct classification rate; LC-OCT, line-field confocal tomography.

## Data Availability

The data presented in this study are available on request from the corresponding author.
